# Expression of the Embryonic Cancer Stem Cells’ Biomarkers SOX2 and OCT3/4 in Oral Leukoplakias and Squamous Cell Carcinomas: A Preliminary Study

**DOI:** 10.7759/cureus.45482

**Published:** 2023-09-18

**Authors:** Vasileios Zisis, Dimitrios Andreadis, Pinelopi A Anastasiadou, Meni Akrivou, Ioannis S Vizirianakis, Lefteris Anagnostou, Dimitrios Malamos, Konstantinos Paraskevopoulos, Athanasios Poulopoulos

**Affiliations:** 1 Oral Medicine/Pathology, School of Dentistry, Aristotle University of Thessaloniki, Thessaloniki, GRC; 2 Pharmacology, Aristotle University of Thessaloniki, Thessaloniki, GRC; 3 Health Sciences, University of Nicosia, Nicosia, CYP; 4 Pharmacy, Aristotle University of Thessaloniki, Thessaloniki, GRC; 5 Oral Medicine, National and Kapodistrian University of Athens, Athens, GRC; 6 Oral and Maxillofacial Surgery, Papanikolaou Hospital, Aristotle University of Thessaloniki, Thessaloniki, GRC

**Keywords:** sox2, oct3/4, oral leukoplakia, oral cancers, cancer stem cells

## Abstract

Introduction: Cancer stem cells (CSCs) are incriminated for initiating the process of carcinogenesis either de novo or through the transformation of oral potentially malignant disorders (OPMDs) to oral squamous cell carcinoma (OSCC). The aim of this study was to detect the expression of embryonic-type CSC markers OCT3/4 and SOX2 in OSCCs and oral leukoplakias (OLs), the most common of OPMDs.

Materials and methods: The study type is experimental, and the study design is characterized as semiquantitative research, which belongs to the branch of experimental research. The experiment was conducted in the Department of Oral Medicine/Pathology, School of Dentistry, Aristotle University of Thessaloniki, Greece. This study focuses on the semiquantitative immunohistochemical (IHC) pattern of expression of CSCs protein-biomarkers SOX2 and OCT3/4, in paraffin embedded samples of 21 OSCCs of different grades of differentiation and 30 cases of OLs with different grades of dysplasia, compared to five cases of normal oral mucosa in both terms of cells’ stain positivity and intensity. Statistical analysis was performed through SPSS 2017 Pearson Chi-square and the significance level was set at 0.05 (p=0.05). The expression of the respective genes of SOX2 and OCT3/4 was studied through quantitative polymerase chain reaction (qPCR), in paraffin-embedded samples of 12 cases of OLs with mild/non dysplasia and 19 cases moderately/poorly differentiated OSCCs(n=19) and five normal mucosa using the Independent Paired T-test.

Results: The genes SOX2 and Oct3/4 were expressed in all examined cases although no statistically significant correlations among normal, OL and OSCC, were established. A nuclear/membrane staining of OCT3/4 was noticed only in three out of 21 OSCCs but in none of OLs or normal cases (without statistical significance). A characteristic nuclear staining of SOX2 was noticed in the majority of the samples, mostly in the basal and parabasal layers of the epithelium. SOX2 was significantly detected in the OSCCs group (strong positivity in 17/21) than in the OL group (30 cases, mostly mildly stained) (p-value=0.007), and the normal oral epithelium (mild stained, p=0.065). Furthermore, SOX2 was overexpressed in well differentiated OSCCs group (5/OSCCs, strongly stained) rather than in mildly dysplastic and non-dysplastic OLs samples (14/OLs, mildly stained) (p-value =0.035).

Conclusion:The characteristic expression of SOX2 but not of OCT3/4 in OLs' and OSCCs’ lesions suggests the presence of neoplastic cells with certain CSC characteristics whose implication in the early stages of oral tumorigenesis could be further evaluated. The clinical use of SOX2, as prognostic factor, requires further experimental evaluation in larger number of samples.

## Introduction

The term 'oral potentially malignant disorder' (OPMD) is attributed to oral mucosal disorders/lesions which exhibit an increased risk for malignant transformation compared to healthy mucosa [1]. The most common OPMD is oral leukoplakia (OL) [2]. On the histological level, OL is typically divided into non-dysplastic, mild, moderate, and severe subtypes [3]. Among them, the moderate and severe OLs reveal a higher risk of cancer transformation [4]. Oral squamous cell carcinoma (OSCC) arises from cells of the stratified squamous epithelium whose microscopic and molecular parameters seem to determine its therapy and prognosis [5]. OSCC can be divided, histologically, into three categories of differentiation: high, moderate, and poor. The moderate and poor differentiation tumors are related to worse prognosis (WHO classification 2017) [6].

When multiple genetic changes affect the oral squamous epithelium, a highly complex multifocal process known as oral carcinogenesis occurs [7]. This process may also begin by a specific population of cancer stem cells (CSCs), which also participate in the formation of tumors. The CSC theory suggests that cells are organized in a hierarchy with CSCs being at the top and normal cancer cells at the bottom [7]. Although there are several markers of CSCs, in general, there are no specific for OSCCs. Markers such as CD44, CD24, CD133, Musashi-1, CD147 and ALDH have occasionally been recommended previously as possible CSC indicators in OSCC, but nowadays, markers such as OCT4, NANOG, and SOX2 express comparable proteins to those that control embryonic stem cells (ESCs) in OSCC [8-15]. The transcription factor OCT4 is a regulator of the Pit-Oct-Unc (POU) domain and plays a critical role in early embryogenesis, maintenance of ESC pluripotency and aberrant cell reprogramming resulting in carcinogenesis [16-19]. SOX2 protein is a SRY-related high-mobility-group (HMG) box transcription factor, involved in multiple signal transduction pathways and thus in normal developmental and many pathological processes including cell proliferation, migration, invasion, stemness, tumorigenesis, anti-apoptosis, and chemoresistance [16-19].

CSCs’ biomarkers have been utilized in the past to identify and distinguish different subgroups of CSCs [20]. OCT4 and SOX2 are considered as embryonic stem cell markers, as they are both, naturally, expressed by embryonic stem cells, may reprogram somatic cells into embryonic stem cell-like states [21,22]. These are the master regulators for self-renewal and maintenance of the stem cell population [23,24]. Therefore, it may be suspected that OCT3/4 and SOX2 markers are expressed in cells having stem cell-like features, like those at the basal layer of the oral epithelium. The upregulation of OCT3/4 and SOX2 could therefore be correlated with the increased risk of malignant transformation and the worse tumor prognosis [17-19]. SOX2 seems to play a key role in the development of cancers of various areas like from breast, colorectum, skin, head, and neck, including the oral cavity as well [25,26].

The aim of this study was to examine the expression of SOX2 and OCT3/4 in normal oral mucosa, OLs and OSCCs lesions of various degrees of dysplasia and differentiation grades, respectively.

## Materials and methods

The paraffin-embedded tissue samples of normal oral epithelium, OLs, and OSCCs, for both immunohistochemical (IHC) and quantitative polymerase chain reaction (qPCR) methods, were derived from biopsies conducted in the period 2009-2019 in the Department of Oral Medicine/Pathology, School of Dentistry, in the Oral and Maxillofacial Surgery Clinic of G. Papanikolaou General Hospital, Aristotle University, and Oral and Maxillofacial Surgery Clinic of St Luke Hospital, Thessaloniki, Greece. The tissues were fixed in a 10% formaldehyde solution, and then were embedded into paraffin for long-term preservation. Multiple 4 μm-thick for immunohistochemistry (IHC) and 10 μm-thick sections for qPCR methods were used. The presence of adequate precancerous or cancerous epithelial tissue (more than 70% per tissue specimen in each section to avoid possible false positivity of non-epithelial cells for the relevant markers) was the main inclusion criterion, for the procedure of qPCR technique. The exclusion criterion included a lack of adequate tissue. The patients were informed and they consented, and the study was approved by the Ethics Committee of the Dental School Aristotle University of Thessaloniki, Greece (Nr 8/03.07.2019).

The study type is experimental, and the study design is characterized as semiquantitative research which belongs to the branch of experimental research. This study examined the IHC pattern of expression of SOX2 and OCT3/4 in tissue samples from 30 cases of OLs and 21 OSCCs in comparison to five cases of normal mucosa (healthy epithelium adjacent to reactive-benign-lesions-such as fibromas), which is the control group. The epidemiological and topographical data of the examined OL cases are summarized in Table 1 and of the examined OSCC cases in Table 2.

**Table 1:** The epidemiological and topographical data of the 30 OL cases (IHC) OL: Oral leukoplakia; IHC: Immunohistochemistry

Patients		Location							General demographic data
		Lip	Corner of mouth	Buccal mucosa	Mucobuccal fold	Gingivae	Palate	Tongue	
Histological differentiation	None-mild	1	1	4		1		7	M:F=1; Males: Median=64, Min.=37, Max.=75; Females: Median=61, Min.=12, Max.=84
	Moderate			1				6	
	Severe			1	2		1	5	
Sex	Males		1	3	1		1	9	
	Females	1		3	1	1		9	
Age	<30					1			
	>30/≤50	1	1					6	
	>50/≤60			2	1			2	
	>60			4	1		1	10	

**Table 2:** The epidemiological and topographical data of the 21 OSCC cases (IHC) OSCC: Oral squamous cell carcinoma; IHC: Immunohistochemistry

Patients		Location				General demographic data
		Lip	Buccal mucosa	Tongue	Floor of mouth	
Histological grade	Well	2		3		M:F=0.75; Males: Median=60, Min.=43, Max.=77; Females: Median=76.5, Min.=43, Max.=82
	Moderate		4	5	1	
	Poor			5	1	
Sex	Males	2	3	4		
	Females		1	9	2	
Age	≤30					
	>30/≤50		1	2		
	>50/≤60	1		3		
	>60	1	3	8	2	

The 30 cases of OL were further divided into two subgroups according to the WHO 2005 binary taxonomy for OLs [27]. The first subgroup included 14 cases of non-dysplastic and mildly dysplastic OLs, whereas the second subgroup included 16 cases of moderately and severely dysplastic OLs. The included 21 cases of OSCCs were initially divided, according to the WHO 2017 OSCC taxonomy, on the degree of histological differentiation, into two subgroups [6]. The first group included the well differentiated (five cases) and the second group the moderate and poorly differentiated OSCC (16 cases). The protocol of the IHC technique included the use of an anti-SOX2 antibody (sc-365823, SantaCruzBiotechnology, Dallas, USA; mouse) and anti-OCT3/4 antibody (sc-5279, SantaCruzBiotechnology, Dallas, USA; mouse), both at a dilution of 1:100. The Dako Envision System Flex+, as secondary stain detection system, and a chromogenic agent application were used according to the manufacturer’s directions (Autostainer, Dako Dab Envision, Denmark). The staining of the same tissue sample with both of the antibodies was not feasible through this technique. The evaluation of IHC staining of SOX2 and OCT3/4 was performed in comparison with standard hematoxylin staining obtained by microscopical examination of the specimens. The examination was performed by two observers (DA and VZ). Positive staining was defined when the nucleus or the intercellular membrane was stained brown in >5% of cells. The total evaluation of the staining of SOX2 and OCT3/4 was defined as histochemical score. This score was obtained by calculating the percentage of positive cells, into a scale of 1-3 (positivity of cells: (1) 6-35%, (2) 36-70%, (3) 71-100%) and then multiplying it by 1 (+, weak staining) or 2 (++, strong staining) according to our previous model [28]. The model used is summarized in Table 3.

**Table 3:** Model of IHC score IHC: Immunohistochemical

Scale 1-3		Score with weak staining (multiply by 1)	Score with strong staining (multiply by 2)
0-5%	0	0	0
6-35%	1	1	2
36-70%	2	2	4
>71%	3	3	6

Especially, in OLs the three-tier scale was defined as follows: 1) Presence of positive cells (6-35%) in one third of the epithelium; 2) presence of positive cells (36-70%) in two-thirds of the epithelium; 3) presence of positive cells (>71%) throughout the epithelium.

Quantitative polymerase chain reaction

In this study the relative expression of the SOX2 gene was examined in tissue samples of 11 OLs without or with mild dysplasia and 18 OSCCs with moderate/poor grade of differentiation (the epidemiological and topographical data of the examined OL cases are summarized in Table 4 and of the examined OSCC cases in Table 5).

**Table 4:** The epidemiological and topographical data of the 11 OL cases (qPCR) OL: Oral leukoplakia; qPCR: Quantitative polymerase chain reaction

Patients		Location								General demographic data
		Lip	Corner of the mouth	Buccal mucosa	Mucobuccal fold	Oral mucosa	Alveolar Mucosa	Tongue	Floor of mouth	
Histological differentiation	None-mild	2	1	3	1	1	1	1	1	M:F=1.75; Males: Median=64, Min.=41, Max.=57; Females: Median=50, Min.=34, Max.=63
Sex	Males	1	1	2	1			1	1	
	Females	1		1		1	1			
Age	>30/≤50	1		2			1		1	
	>50/≤60					1		1		
	>60	1	1	1	1					

**Table 5:** The epidemiological and topographical data of the 18 OSCC cases (qPCR) OSCC: Oral squamous cell carcinoma; qPCR: Quantitative polymerase chain reaction

Patients		Location						General demographic data
		Retromolar fossa	Floor of the mouth	Buccal mucosa	Mucobuccal fold	Alveolar mucosa	Tongue	
Histological grade	Moderate	1		2	2	3	7	M:F=0.80; Males: Median=61, Min.=30, Max.=80; Females: Median=76, Min.=27, Max.=86
	Poor		1			2		
Sex	Males	1		2		3	2	
	Females		1		2	2	5	
Age	≤30	1					1	
	>30/≤50					1	1	
	>50/≤60			1		1	1	
	>60		1	1	2	3	4	

This technique looked for the genes’ expression in order to confirm the consequent presence of protein products in the cell membrane or and within the nucleus. The QiagenQuantiTectPrimerAssay(200) Hs_QSOX2_1_SGQuantiTectPrimerAssay, Qiagen, Hilden, Germany) and OCT3/4 gene (CustomOCT3/4 EurofinsMWG, Qiagen, Hilden, Germany) were used through qPCR.

The deparaffinization of the obtained tissues was performed using the deparaffinization solution (QiagenDeparaffinization Solution, Qiagen, Hilden, Germany, #73504). The RNA extraction was accomplished using RNeasy FFPE Kit (QiagenRNeasyFFPEKit, Qiagen, Hilden, Germany) according to the manufacturer’s recommendations. DNase treatment was also performed to eliminate genomic DNA. Spectrophotometry was conducted to evaluate the quality and quantity of extracted RNA (Epoch, Biotek, Vermont, USA). Extracted RNA was prone to synthesize complementary DNA (cDNA) usingQiagen kit according to the manufacturer’s protocol. 50 nanograms of total RNA was used as template to generate first-strand cDNA using cDNA kit (QiagenQuantiTectReverse TranscriptionKit, Qiagen, Hilden, Germany, #205311). PCR was carried out on 7,500 Real Time PCR System (Applied Biosystems, Foster City, CA,USA) using KAPA SYBR FAST qPCR Master Mix kit (#KK4602) in a total volume of 10 μl with the following thermal cycling parameters: 95°C for three minutes, followed by 40 cycles of denaturation at 95°C for three seconds and annealing/extension at 60°C for 60 seconds. All reactions were carried out in triplicates and the resulting text file was exported to Microsoft excel. The expression of the genes were normalized to the average Ct value of β-actin(Qiagen QuantiTectPrimerAssay (200) Hs_ACTB_1_SGQuantiTectPrimerAssay, Qiagen, Hilden, Germany). The DNA sequences of the primers are summarized in the Table 6.

**Table 6:** The DNA sequences of the primers implemented. The mean expression of the gene under investigation was normalized by comparison to the mean expression of βactin. In particular, ΔCt was calculated (Ct mean of SOX2 minus the Ct mean of βactin, Ctmean ofOCT3/4 minus the Ctmean of β actin).

Primers	DNA sequence
SOX2	NM_003106
OCT3/4	F:AGTGAGAGGCAACCTGGAGA
	R:CAAAAACCCTGGCACAACT
βactin	F:TTG-CTG-ACA-GGA-TGC-AGA-AG
	R:TGA-TCC-ACA-TCT-GCT-GGA-AG

Statistical analysis

Statistical analysis was performed through the SPSS software (2017, IBM SPSS Statistics for Windows, Version 25.0, IBM Corp. Armonk, USA) with Pearson Chi-square test and the Fisher’s Exact test depending on the sample size and the significance level was set at 0.05 (p=0.05) regarding the IHC whereas independent Paired T-test was implemented, and the significance level was set at 0.05 (p=0.05) for the qPCR.

## Results

OCT3/4 and SOX2 genomic profile

All the samples(normal, OL and OSCC)were positive, regarding the gene expression of both OCT3/4 and SOX2, although no statistically significant correlations were established between the OL (see Table 7) and OSCC tissues (see Table 8) in relation with their genomic profile.

**Table 7:** Gene expression of OCT3/4 and SOX2 in 11 OL cases All samples were positive in qpcr for OCT3/4  and SOX2 but no statistical correlation differences were found among OL, OSCCs (p>0.05), OCT3/4 (p= 0.378), and SOX2 (p=0.995). OL: Oral leukoplakia; qPCR: Quantitative polymerase chain reaction

Location	Histology (Dysplasia)	Patient		qPCR	
		Age	Sex	Δct	
				OCT3/4	SOX2
Mucobuccal fold	None	66	Male		8.04
Lip-upper	None	41	Male	15.18	8.305
Lip-lower	None	63	Female	-4.265	
Alveolar mucosa	None	34	Female	-1.8216	-2.695
Tongue	Mild-	57	Male	-0.125	
Oral Mucosa	Mild	60	Female	-2.2483	-6.005
Buccal mucosa	Mild	69	Male	-0.485	0.523
Corner of mouth	Mild	61	Male		-5.81
Buccal mucosa	Mild	42	Male	3.635	
Buccal mucosa	Mild	40	Female	0.74	0.5
Floor of mouth	Mild	47	Male	-0.215	

**Table 8:** Gene expression of OCT3/4 and SOX2 in 18 OSCC cases All samples were positive in qpcr for OCT3/4  and SOX2 but no statistical correlation differences were found among OL, OSCCs (p>0.05), OCT3/4 (p= 0.378), and SOX2 (p=0.995). OSCC: Oral squamous cell carcinoma; qPCR: Quantitative polymerase chain reaction

Location	Histology (Grade)	Patient		qPCR	
		Age	Sex	Δct	
				OCT3/4	SOX2
Tongue	Moderate	51	Male	4.005	
Buccal mucosa	Moderate	63	Male	-1.2	-5.65
Alveolar mucosa	Poorly	80	Male	-0.41	
Alveolar mucosa	Moderate	59	Female	-3.115	-5.375
Buccal mucosa	Moderate	59	Male	-1.865	- 0.63
Alveolar mucosa	Moderate	85	Female	3.8	0.84
Tongue	Moderate	63	Female	11.625	3.41
Tongue	Moderate	79	Female		3.895
Tongue	Moderate	86	Female	6.465	
Mucobuccal fold	Moderate	77	Female	-0.45	-5.995
Mucobuccal fold	Moderate	75	Female	3.75	1.375
Tongue	Moderate	67	Male	3.59	6.745
Tongue	Moderate	42	Female	10.795	9.89
Tongue	Moderate	27	Female	7.025	-0.785
Alveolar mucosa	Moderate	72	Male	-1.4766	
Retromolar fossa	Moderate	30	Male	3.225	-1.595
Alveolar mucosa	Poorly	35	Male		-1.015
Floor of mouth	Poorly	82	Female	4.7166	

In certain cases, qPCR was not carried out successfully due to insufficient tissue quantity (four cases for OCT3/4, nine cases for SOX2).

IHC staining for OCT3/4 and SOX2

The next tables summarize the results of IHC staining (localization, intensity pattern, statistical correlations) for OCT3/4 and SOX2 in normal oral epithelium (see Table 9), OL (see Table 10), and OSSCs (see Table 11). 

**Table 9:** Results of IHC staining for OCT3/4 and SOX2 in 5 normal oral epithelium cases IHC: Immunohistochemical

Score of OCT3/4			Location of OCT3/4	Level of expression of OCT3/4	Score of SOX2					Location of SOX2	Level of expression of SOX2
0	1	2	-	-	0	1	2	3	4	Nuclear	A few basal cells only
5						5					

**Table 10:** Results of IHC staining for OCT3/4 and SOX2 in 30 OL cases Statistical analysis (only for SOX2): Statistically significantly lower expression in OLs vs OSCCs (p= 0.007), OL (non- and mildly dysplastic) showed statistically significantly lower expression vs poorly and moderately  differentiated OSCCs (0.003) and well-differentiated OSCCs (0.035). OL: Oral leukoplakia; OSCC: Oral squamous cell carcinoma; BMZ: Basement membrane zone; IHC: Immunohistochemical

Histological characteristics	Score of OCT3/4	Location of OCT3/4	Level of expression of OCT3/4	Score of SOX2			Location of SOX2	Level of expression of SOX2
	0			1	2	4		
Non + mild Dysplasia	14	-	-	8	6		Nuclear in basal parabasal and spinous cells	+
Moderate + severe Dysplasia	16	Nuclear (basal)+membranous (BMZ) in 2 dysplastic areas close to 1 poordif. OSCC and 1 moderdif. OSCC	-	10	3	3	Nuclear in basal parabasal and spinous cells	++ Increased with the severity of dysplasia

**Table 11:** Results of IHC staining for OCT3/4 and SOX2 in 21 OSCC cases Statistical analysis (Only for SOX2): Statistically significantly lower expression in OLs vs OSCCs (p= 0.007), OL (non- and mildly dysplastic) showed statistically significantly lower expression vs poorly and moderately  differentiated OSCCs (0.003) and well-differentiated OSCCs (0.035). OL: Oral leukoplakia; OSCC: Oral squamous cell carcinoma; BMZ: Basement membrane zone; IHC: Immunohistochemical

Histological characteristics	Score of OCT3/4			Location of OCT3/4	Level of expression of OCT3/4	Score of SOX2				Location of SOX2	Level of expression of SOX2
	0	1	2			0	1	2	4		
Well	5			-	-		1	2	2	Nuclear in basal parabasal and spinous cells	++
Moderate + poor	13	2	1	Nuclear (basal) and membranous (BMZ) in 1 moderate and 2 poor OSCCs	+	4	6	1	5	Nuclear in basal parabasal and spinous cells	++ Increased with the grade of differentiation

A possible association of the staining pattern of the above CSCs biomarkers with characteristics like patients’ age, sex, habits (alcohol/smoking); lesions’ location, type, response to treatment, or even metastases; and final outcome were difficult to be evaluated statistically due to missing information from such a small group of patients.

OCT3/4 staining

**Figure 1:**
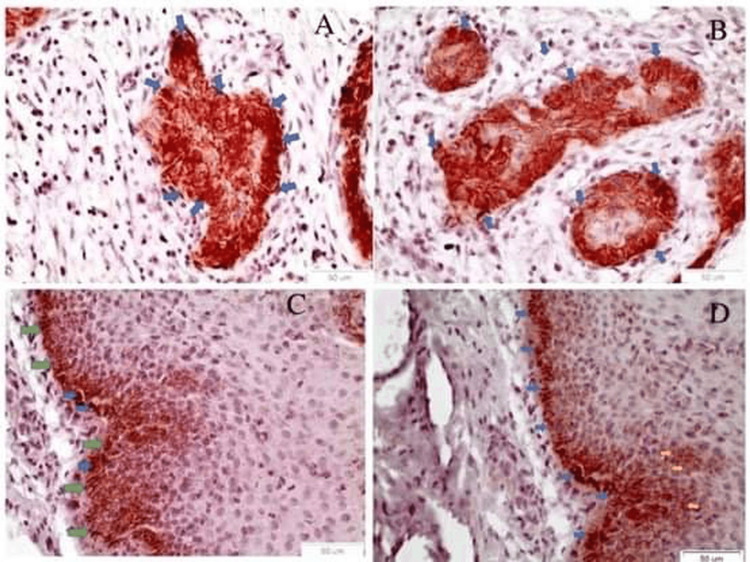
OCT3/4 IHC staining A) A cancerous nest in a moderately differentiated OSCC comprised of cancer cells positive to OCT3/4 (blue arrows) (Χ40); B) Cancerous foci are noticed, with cancer cells positive to OCT3/4 (blue arrows) in poorly differentiated OSCC (Χ40); C) Adjacent to moderately differentiated OSCC, the dysplastic epithelium exhibits the typical membrane staining of the interface area between the basal layer and the basal membrane  zone (green arrows) (Χ40). Nuclear staining is also noticed in individual cells (blue arrows); D) Adjacent to moderately differentiated OSCC, the dysplastic epithelium exhibits the typical membrane staining of the interface area between the basal epithelial layer and the basal membrane (blue arrows) (Χ40). Membrane staining is also noticed in individual cells at the parabasal layer (yellow arrows). OSSC: Oral squamous cell carcinoma; IHC: Immunohistochemical

OCT3/4 was detected on both membranous and nuclear areas in one case of a moderately differentiated (Figure 1A) and in two cases of poorly differentiated (Figure 1B) OSCCs. Importantly, in the adjacent dysplastic epithelium of the two positive OSCC cases, the biomarker OCT3/4 exhibits a nuclear (Figure 1C) as well as a membrane pattern of staining at the interface zone between the basal cell layer and the basement membrane (Figure 1D).

SOX2 staining

**Figure 2:**
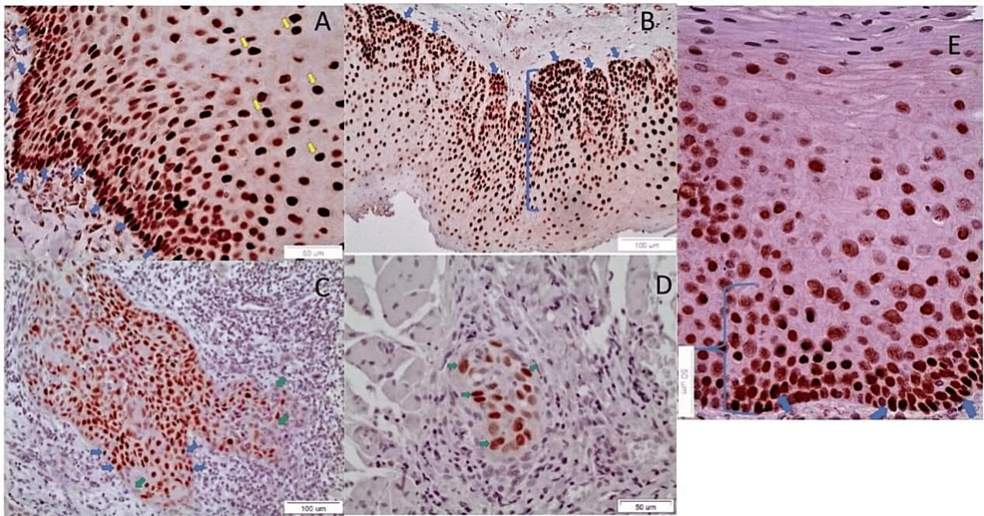
SOX2 IHC staining A) The nuclear staining of SOX 2 is noticed, mostly in the lower of the epithelium, in a case of non-dysplastic OL, characteristically, at the interface area between the basal epithelial layer and the basal membrane (blue arrows). Scattered, positively stained, cells are noticed also in the prickle layer (yellow arrows) (X40); B) The nuclear staining of SOX2 is noticed in the lower twp-thirds of the epithelium (blue bracket) in a case of moderately dysplastic OL (X20). Typical nuclear staining at the basal cells is noticed, at the interface area between the basal epithelial layer and the basal membrane (blue arrows); C) A well-differentiated OSCC manifests cancerous foci with small cells positive to SOX2 (blue arrows) (Χ20). Lack of staining is also noticed in scattered peripheral cancer cells (green arrows); D)  A cancerous focus is noticed with SOX2 positive CSCs (green arrows), among muscle cells, in a moderately differentiated OSCC (Χ40); E) The nuclear staining of SOX 2 (blue arrows) is sporadically noticed in the lower third of the epithelium (blue bracket), in one case of normal oral mucosa (X40). OL: Oral leukoplakia OSSC: ; IHC: Immunohistochemical; CSC: Cancer stem cell

On the other hand, SOX2 revealed only a nuclear pattern of expression, in all of the samples, in the basal, parabasal and spinous cells. The quantitative/qualitative pattern of expression was increased in parallel with the severity of dysplasia of OLs (Figure 2A: no dysplasia/mild dysplasia OL group, Figure 2B: moderate/severe dysplasia OL group), as well as the grade of differentiation of OSCCs (Figure 2C: well-differentiated OSCC group, Figure 2D: moderate/low differentiation OSCC group) in comparison with its occasional presence at the basal cell layer of the normal oral epithelium (Figure 2E).

The expression of SOX2 was statistically significant higher in OSCCs than in OLs (Pearson chi-square, p-value=0.007). OL without dysplasia or with mild dysplasia showed a statistically significant lower expression of SOX2 than in poorly/moderately (Fisher’s exact test, p-value=0.003) and well differentiated OSCCs (Pearson chi-square, p-value=0,035). Well differentiated OSCCs expressed SOX2 at statistically significant higher level than in normal oral mucosa (Fisher’s exact test, p-value=0,048)

In contrast, there was no statistically significant difference in the expression of SOX2 between the moderately and severely dysplastic OLs, as well as between the well differentiated OSCC and the moderately and poorly differentiated OSCC. SOX2 was significantly more intense in moderately and severely dysplastic OLs and the OSCCs than in mild/non-dysplastic OLs and normal oral epithelium.

## Discussion

CSCs seem to play an important role in the initiation and development of various malignancies [29-31]. The detection of CSCs in solid cancers was firstly confirmed in 2003, by Al-Hajj et al., who showed that only a fraction of CD44+/CD24+ or negative breast cancer cells could create a tumor with the same characteristics of the initial tumor [32]. Since then, a few studies have pointed out the role of CSCs in the initiation, development, advancement, and recurrence of cancer as well as in the resistance in chemo and/or radiotherapy by inhibiting cell death and enhancing cell-dormancy [9,33-37]. Therefore, CSCs may be involved in the development of OSCC [29]. A small portion of the oral cancer cell population possesses characteristics similar to those of CSCs [10]. OSCC neoplastic cells’ islands are composed of a mixture of differentiated cells (that are unrelated to tumor proliferation), transitory proliferating cells and few cells with the capacity for abnormal cell division and self-renewal, known as CSCs) [10]. CSCs in OSCCs may have originated from normal oral epithelial stem cells, which are located among the basal cells of stratified squamous epithelium [38]. Normal oral epithelial stem cells support the physiological tissue renewal, and their differentiation provides the upper epithelial cell elements [30]. OCT4 plays a crucial role in early embryogenesis, maintenance of ESC pluripotency and aberrant cell reprogramming [16,17]. In addition, OCT4 gene is also linked to oncogenesis: tumor transformation, tumorigenicity, invasion, and metastasis of OSCC by playing a role in the regulation of epithelial-mesenchymal transition (EMT) [23,39,40]. OCT3/4 expression is related to neck metastasis (DNM) by enhancing cancerous cell motility and invasiveness [41]. On the other hand, the SOX2 protein seems to participate in cell proliferation, migration, invasion, stemness, tumorigenesis, anti-apoptosis, and chemoresistance [18,19]. SOX2 is expressed within the tumor nests, the peri-tumor stroma and microvessels [42]. SOX2 expression is correlated with smaller size, and early tumor stage, and longer disease-free survival rate in OSCCs [43]. However, in our study, one out of three dead patients with tongue lesions with positive lymph nodes and without metastasis had negative staining for both markers, contradicting the association between SOX2 expression and better clinical situation (this association must remain under consideration since it is supported by a very limited number of patients). Silencing SOX2 suppresses the expression of drug resistance and anti-apoptotic genes and increases the sensitivity of the cells to radiation-combined-cisplatin chemo therapy [19]. According to Grubelnik et al. Nanog and OCT4 genes were overexpressed in cancers with lymph node metastasis compared to cases without metastases [44]. In our study, the qPCR experiment showed that all OLs and OSCCs revealed SOX2 and OCT3/4genes but failed to establish any statistically significant correlation among them. The present study is the first to examine the expression of the above CSCs biomarkers in the most common OPMD lesion, OL (ranging from non dysplastic OL to mildly, moderately and severely dysplastic OL). Thus, it is the first study to investigate the differences in expression of these markers in relation to the degree of dysplasia of leukoplakias and differentiation of oral carcinomas. Although this is a preliminary study, its results showed that SOX2 expression pattern is increased with the degree of dysplasia reaching its highest threshold in the moderate/severe dysplasia OL. This finding could suggest that the presence of SOX2 in OLs without or mild dysplasia may constitute a possible predictor marker for unfavorable prognosis in the future. In contrast, OCT3/4 staining was noticed only rarely in OSCCs having no prognostic value. SOX2 and OCT3/4 manifest different pattern of expression since the first precipitates the further development of dysplasia in OLs whereas the latter is expressed in already relatively undifferentiated OSCCs representing an already more aggressive phenotype. Human papillomavirus (HPV) infection, gene polymorphisms, or the presence of lymph nodal metastasis may affect the distribution pattern of embryonic cell markers and they consist possible future perspectives for research [45].

Limitations

The limitations of our study included the lack of follow-ups of the patients from whom the tissue specimens were derived, the lack of tumor-node-metastasis (TNM) classification and of the HPV status regarding the OSCCs included.

## Conclusions

Our findings imply that SOX2 and OCT3/4 expression differs as SOX2 comes higher as the dysplasia increases while OCT3/4 is found stable in OSCCs only. New studies should be undertaken to investigate further the role of these two markers in OL and OSCCs and in comparison with additional new biomarkers in even larger patient samples using immunohistochemistry and qPCR in an attempt to better understand the nature and role of CSCs during carcinogenesis. Ideally, these patient samples should include tissue from OL, OSCC, lymph nodal metastasis, and distant metastasis.
